# Use of GnRH Agonist in Dogs Affected with Leishmaniosis

**DOI:** 10.3390/ani11020432

**Published:** 2021-02-07

**Authors:** Michela Pugliese, Vito Biondi, Marco Quartuccio, Santo Cristarella, Giovanni Emmanuele, Gabriele Marino, Luigi Liotta, Annamaria Passantino

**Affiliations:** 1 Department of Veterinary Sciences, University of Messina, 98168 Messina, Italy; michela.pugliese@unime.it (M.P.); vbiondi@unime.it (V.B.); santo.cristarella@unime.it (S.C.); marinog@unime.it (G.M.); luigi.liotta@unime.it (L.L.); annamaria.passantino@unime.it (A.P.); 2 BIOGENE Veterinary Diagnostic Center, Via Giacomo Leopardi, 50, 95127 Catania, Italy; giovanniemmanuele@tiscali.it

**Keywords:** canine leishmaniosis, deslorelin acetate, testosterone

## Abstract

**Simple Summary:**

Most parasitic diseases, including *Leishmania* infection, often show more severe clinical signs in males than females. This is related to a different immune response, presumably related to testosterone activity. The present study aimed to evaluate the efficacy of a gonadotropin-releasing hormone GnRH agonist implant (deslorelin acetate) in association with meglumine antimoniate plus allopurinol in the treatment of dogs affected by *Leishmania.* Deslorelin acetate is a GnRH agonist, widely used for chemical reversible sterilization in male dogs, inducing testosterone suppression. Dogs treated with deslorelin show a significant decrease of clinical scores and serological test, suggesting a possible employ of GnRH agonist in the treatment of canine leishmaniosis.

**Abstract:**

Sex-associated hormones such as testosterone have been demonstrated to modulate immune responses, which can result in different disease outcomes. The present study was aimed at evaluating the efficacy of a gonadotropin-releasing hormone GnRH agonist implant as deslorelin acetate in association with meglumine antimoniate plus allopurinol in dogs with canine leishmaniosis (CanL). Twenty-two dogs with CanL confirmed by clinical findings and laboratory tests were included in the study. Dogs were randomized into two groups. A control group (CTR, *n* = 12) was treated with meglumine antimoniate 50 mg/kg SC q 12 h for 28 days plus allopurinol at 10 mg/kg PO q 12 h for the whole study period (six months). An experimental group was treated with allopurinol and meglumine antimoniate, plus an implant of 4.7 mg deslorelin acetate (DES, *n* = 10). The animals were observed for three months, during which clinical evaluation, indirect fluorescent antibody test (IFAT) titre and testosterone assay were performed on time at day (D)0, 90 and 180. A significantly lower clinical score was recorded in DES than in CTR (*p* < 0.01) at D90 and D180 (*p* < 0.01). After 180 days of treatment (D180), a significant reduction of mean levels of IFAT was observed in the DES group (*p* = 0.03). A highly significant reduction of testosterone (*p* = 0.01) was observed in the DES group during the study. No statistical correlation between clinical scores, IFAT titres and testosterone within two groups was observed. Data suggested that the agonist of GnRH may be useful in the treatment of CanL.

## 1. Introduction

Canine leishmaniosis (CanL) is a zoonosis caused by the protozoan *Leishmania infantum* currently considered endemic in southern Europe, Africa, Asia and South America [1,2,3,4]. The disease is transmitted by the bite of sandflies of the genus *Phlebotomus* or *Lutzomyia* in the Old and New World, respectively [5]. The spectrum of clinical and clinicopathological findings is wide-ranging from subclinical and self-limiting to severe disease and is related to the predominant response of the immune system [6,7,8,9,10,11]. The immune response plays an important role in the progression and outcome of disease [12]. Moderate/several clinical forms are associated with an exuberant humoral response mediated by type 2 T helper cell (Th2), while the protective immune response is mediated by T helper cell (Th1) and is correlated with disease resolution [11,12,13]. It has been reported that different factors not related to the immune system may be involved in the effectiveness of immune response and the development of CanL [14]. Sex hormones such as androgens, oestrogens and progestins may have a critical role in defining the intensity of parasite infection by modifying the interplay between the parasite and definitive host. Indeed, receptors for sex hormones are present on the surface of macrophages and T cells, the two major cell types involved in the progression of the disease. It has been hypothesized that testosterone has immunosuppressive effects [15]. Moreover, sex hormones oestrogen and progesterone promote the Th2 immune response, while testosterone seems to favour type Th1 immune response [16]. Gonadotropin-releasing hormone GnRH agonist slow-release implants are widely considered to be a reversible alternative to surgical neutering on males [17]. The action of a GnRH agonist is related to the desensitization of receptors to GnRH, which results in a temporary long-term downregulation of testicular endocrine function in male dogs [18,19]. Given the possible role of testosterone in *Leishmania* spp. infection, the authors have evaluated the effect of treatment with GnRH agonist (deslorelin acetate implant) in association with meglumine antimoniate plus allopurinol on dogs affected by CanL in reducing clinical signs of disease and indirect fluorescent antibody test (IFAT) titre.

## 2. Materials and Methods

### 2.1. Animals

Client-owned unaltered male dogs admitted to the Veterinary Teaching Hospital of the University of Messina were enrolled. Inclusion criteria were an anti-*Leishmania* antibody titre higher than 1/320 in IFAT (cut off 1:80) [20] and cytological identification of *Leishmania* amastigotes or detection of parasite DNA using real-time polymerase chain reaction (RT-PCR), associated with clinical signs and/or laboratory abnormalities corresponding to LeishVet CanL clinical stages [6]. Vaccinated dogs were excluded as well as they have received in at least the 3 months before previous treatments allopurinol, meglumine antimoniate, miltefosine, domperidone, ciclosporin or glucocorticoids or special diet or dietary supplements to improve the immune response. Furthermore, all dogs included in the trial were tested against ehrlichiosis *(Ehrlichia canis*) and babesiosis (*Babesia canis*) using serological tests (IFAT) and microscopic examination in order to exclude co-infections with other vector-transmitted diseases that produce the same clinical signs.

### 2.2. Ethical Statement

The study was conducted according to the Italian laws (D.lgs. 26/2014) and the standards recommended by the European Council Directive 2010/63/EU. It was approved by the Ethics Committee of the Department of Veterinary Sciences, University of Messina (Approval number: 22; date of approval 9 June 2018). All dog owners were informed of the study objectives and the clinical procedures. A written informed consent prior to enrolment was obtained.

### 2.3. Clinical Evaluation

The clinical evaluation (including body weight) was performed before the treatment (D0), at 90 and 180 days (D90 and D180, respectively) using a physician-based scoring system considering sixteen clinical signs. Clinical assessment of the severity of signs attributable to *Leishmania* infection was conducted by an investigator blind to the experimental group. Scores for each variable were added to obtain a maximum of twenty-nine points (Table 1) [6].

### 2.4. Samples Collection

Blood samples were collected from the jugular or cephalic vein and placed in EDTA, heparin lithium or dry tubes depending on the parameter to be analysed. Blood samples collected in dry tubes were allowed to clot for 30 min at room temperature (20–25 °C) and centrifuged (ALC 4235 A ALC Int SRL, Milan, Italy) at 3000 rpm for 20 min. Serum samples obtained after centrifugation were divided into aliquots and stored at −20 °C until analysis. A sample of the first urine in the morning was taken by cystocentesis for urinalysis. All dogs were tested for *Leishmania* by PCR on fine-needle aspirates of bone marrow and/or lymph nodes before the treatment. Furthermore, for direct visualization of the parasite, bone marrow and/or lymph nodes smears were prepared.

### 2.5. Laboratory Testing

A complete haematological, biochemical and urine profile was carried out. The IFAT was performed according to the Office International des Epizooties proposal [21]. *L. infantum* promastigotes (strain MHOM/IT/80/IPT1) were used as a whole-parasite antigen fixed on multi-spot slides (Bio Merieux Spa, Florence, Italy). Canine serum samples diluted in phosphate-buffered saline (1:80) were added on the multi-spot slides with *L. infantum* antigen and incubated in a moist chamber at 37 ± 2 °C for 30 min. Fluorescein labelled anti-canine gamma globulin (Sigma Aldrich, Milan, Italy) was used as conjugate. Two-fold serial dilutions of canine sera were performed to determine the antibody titre. The real-time PCR analysis of fine-needle aspirates of bone marrow and/or lymph nodes was performed using the Illustra Blood genomic Prep Mini Spin kit (GE Healthcare, Milan, Italy) following the instructions of the manufacturer. Serum testosterone concentrations were assayed using an immunoassay system based on the Enzyme Linked Fluorescent Assay (ELFA) principles by Mini VIDAS system (BioMerieux S.A., Lyon, France).

Haematological, biochemical, hormonal, serological, parasitological tests and urinalysis were performed in each dog at three time points: on the day of diagnosis (D0), at D90 and at the end of the study period (D180).

### 2.6. Treatment Protocol

Dogs included were randomly divided into two groups. Dogs in the control (CTR) group received meglumine antimoniate 50 mg/kg SC q 12 h for 28 days, plus allopurinol at 10 mg/kg PO q 12 h for the whole study period (6 months) [21]. Dogs in the experimental (DES) group were treated with allopurinol (10 mg/kg PO q 12 h for 6 months) and meglumine antimoniate (50 mg/kg SC q 12 h for 28 days) [22] plus an implant of 4.7 mg deslorelin acetate (Suprelorin^®^, Virbac, Carros Cedex, France), applied once per time (suppressing the testosterone for 6 months) in the inter-scapular region.

### 2.7. Statistical Analysis

Statistical analysis was performed using SPSS for Windows package (version 17.0, SPSS, Inc., Chicago, IL, USA). Mean and standard deviation of clinical signs, testosterone and IFAT titres were calculated at D0, D90 and D180. The normal distribution of parametric data was evaluated using the D’Agostino–Pearson test. Mann–Whitney was used for non-normally distributed data to assess statistically significant differences in the mean of clinical scores, testosterone and IFAT titres. For the determination of the relationship between variables, Spearman’s rank test was used. Significance was set at *p* ≤ 0.05.

## 3. Results

**Figure 1:**
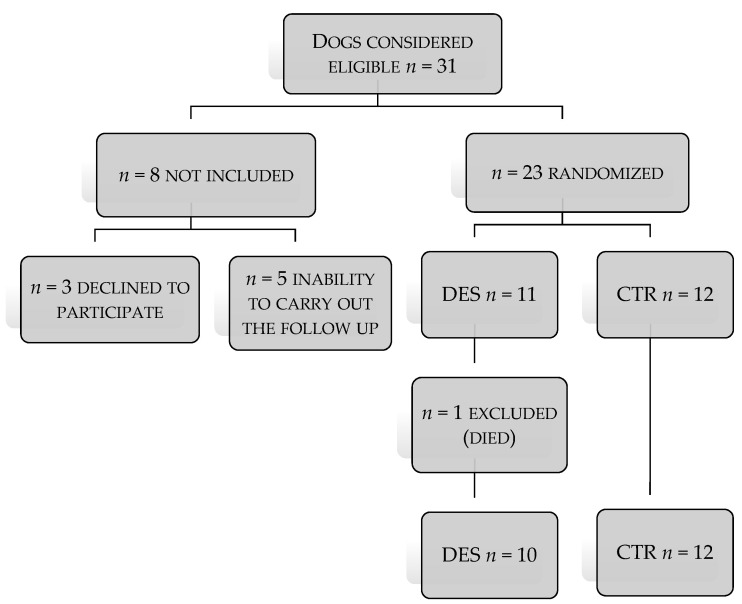
Flow diagram of different phases of the study in group treated plus an implant of 4.7 mg deslorelin acetate (DES) and group control (CTR).

A total of 31 dogs were assessed for eligibility in the study. Of the dogs assessed, only 23 presented inclusion criteria and were randomized into two groups (DES group: *n* = 11, CTR Group: *n* = 12) as reported in Figure 1.

One dog in the DES group died of a cardiorespiratory arrest related to chronic heart failure; two dogs in the CTR group did not complete the study for a reason unrelated to the progression of the disease (noncompliance of owners to carry on at the follow-up visits). A total of 10 dogs belonging to DES group and 12 of the CTR group completed the clinical trial. Dogs’ breeds included in the DES/CTR groups were as follows: Pit-bull (1/2), German Shepherd (1/0), Labrador Retrievers (2/1), crossbreed (2/6), Argentine Dogo (0/1), Jack Russell (2/1), Hound (1/0), Beagle (1/0), English Setter (0/1). Baseline characteristics of the study population are provided in Table 2.

No adverse effects were recorded in the DES. At the baseline, there were no differences between DES and CTR regarding mean age, clinical scores, temperature, body weight and testosterone values (Table 2).

No differences were recorded in clinical scores, laboratory examination, urinalysis and serological test in the two groups initially matched. At the baseline, 2/10 (30%) dogs of DES were in IIa LeishVet CanL clinical stage, 5/10 (50%) were in IIb, 3/10 (30%) were in stage III. Whereas 2/12 (16.6%) of CTR were in stage IIa, 4/12 (33.3%) were in stage IIb and 6/12 (50%) in stage III [6].

**Figure 2:**
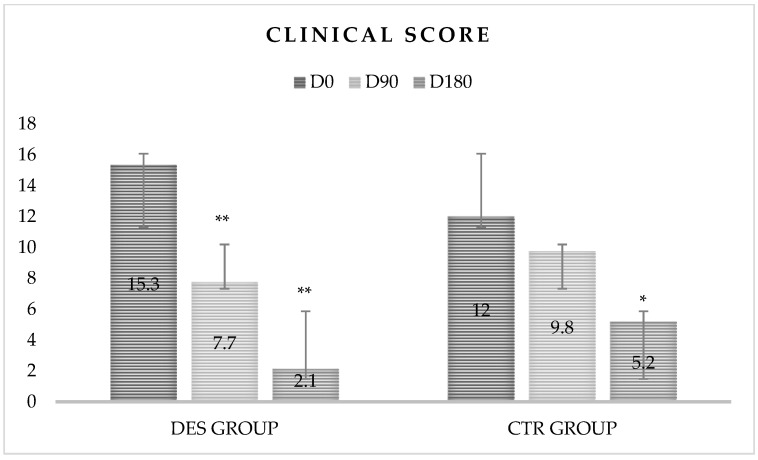
Changes in clinical score in two groups at D0, D90 and D180. Data reported as mean ± Scheme 0. * *p*-Value ≤ 0.05; ** *p*-Value ≤ 0.01

After 180 days of deslorelin acetate implantation a significant lower clinical score was observed in the DES between D0 vs D90 (15.3 ± 6.3 vs 7.7 ± 5.9; *p* = 0.03), D0 vs D180 (15.3 ± 6.3 vs 2.1 ± 4.3; *p* < 0.01), D90 vs D180 (7.7 ± 5.9 vs 2.8 ± 4.3; *p* < 0.01). A significantly lower clinical score in the CTR was observed between D90 vs D180 (9.8 ± 4.3 vs 5.2 ± 4.1; *p* = 0.05), D0 vs D180 (12 ± 4.9 vs 5.2 ± 4.1; *p* = 0.02). No statistical differences were present in the CTR between D0 vs D90 (12 ± 4.9 vs 9.8 ± 4.3; *p* = 0.09). A significantly lower clinical score was recorded in DES than in CTR at D90 and D180 (7.7 ± 5.9 vs 9.8 ± 4.3; *p* < 0.01 and 2.8 ± 4.3 vs 5.2 ± 4.1; *p* < 0.01, respectively) (Table 3; Figure 2).

**Figure 3:**
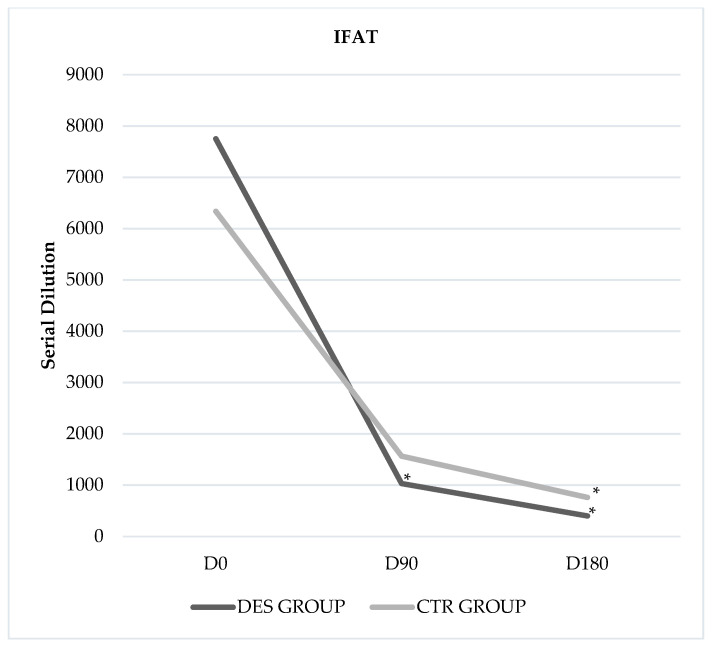
Changes in indirect fluorescent antibody test (IFAT)-determined antibody titres against *Leishmania* in two groups. * *p*-value ≤ 0.05; ** *p*-value ≤ 0.01

A significant reduction in mean levels of *Leishmania* antibody detected by IFAT was observed in the DES between D0 vs D90 (7751 ± 9965 vs 1031 ± 1565; *p* = 0.05), D0 vs D180 (7751 ± 9965 vs 400 ± 814; *p* = 0.04) and D90 vs D180 (1031 ± 1565 vs 400 ± 814; *p* = 0.03). When groups were compared, a significantly lower mean of antibody titre was recorded in DES at D180 (400 ± 814 vs 760 ± 749; *p* < 0.01) (Figure 3).

A high significant reduction of testosterone was observed in DES at D0 vs D90 (3.8 ± 1.6 vs 0.18 ± 0.45; *p* = 0.01) and D0 vs D180 (3.8 ± 1.6 vs 0.17 ± 0.40; *p* = 0.01). No significant changes of testosterone in CTR were recorded over time.

Despite the above-reported changes and significant results, no statistically significant correlation was present between testosterone, clinical score and IFAT titres within the two groups.

## 4. Discussion

The mechanisms relating to the healing process of *Leishmania* infection are still unclear and poorly documented, although some studies have suggested a crucial role in the Th2 response, linked to a deficit in the production of IFN-γ and IL-12, and consequently to an increased susceptibility to infection [23]. However, whatever the specific processes regulating the different types of immunological response in *Leishmania* spp., it would appear that sex hormones influence the immune response, making males more susceptible to disease [24].

The main goal of this study was to evaluate the possible effectiveness of deslorelin implantation associated with meglumine antimoniate plus allopurinol in the treatment of CanL.

Deslorelin acetate is a GnRH superagonist, widely accepted as an alternative to surgical neutering to induce temporary infertility in healthy, intact, adult male dogs and ferrets [17,18,19,20,21,22,23,24,25]. After the application, an initial increase of hormones in pituitary gland has observed, which determines desensitization of the receptors responsible for the gonadotrophin release, followed by the depletion in luteinizing hormone and follicle-stimulating hormone, accompanied by a notable reduction in androgens production [26]. For dogs, the duration of efficacy of the 4.7 mg deslorelin GnRH agonist has been reported to be a minimum of 180 to 400 days for dogs [17].

Dogs treated with deslorelin showed a clinical improvement; the mean clinical score reduction was of 49.3% at D90 and of 81.7% at D180, while group control showed a mean clinical score decrease of 24.1% at D90 and 43% at D180. In agreement with different authors, the reduction of clinical signs could reduce the sandflies’ infectivity [2,26,27]. Similar to what was reported in this study, in an experimental study carried out in mice, the orchiectomy resulted in greater resistance in males than females against *Leishmania major* [28]. In contrast, female mice experimentally infected with *Leishmania mexicana* have been reported to be more resistant to infection [29,30], suggesting that the greater predisposition of males than females to develop the disease would be due to the ability of the latter to produce IFN-γ through a Th1-type response [31,32,33]. Different interactions between genotype, sex-dependent differences and species-specific responses during infection with *Leishmania* spp. (*L. major* and *L. tropica*) on a mouse model are described. Clinical signs observed in mice females experimentally infected are milder, not related to parasitic load, conversely to males where the clinical involvement was more severe and related to increase of inflammatory mediators [31]. A study carried out in male mice treated with oestrogen and females treated with testosterone has reported lesions following *L. panamensis* infection more pronounced in animals treated with testosterone than in those treated with oestrogen [32]. Furthermore, lesions due to *L. panamensis* developed only in adult males who had reached biological maturity [32].

Similarly, data collected in human population also show that males develop skin manifestations more frequently than females [33,34]. Johannsen et al. [35] have reported a higher incidence in males less than one year of age than females, presumably related to a transient postnatal increase in steroid hormone levels observed in male infants. Overall data show that the prevalence of cutaneous leishmaniasis in males increases in puberty, reaching its highest levels in adulthood, and decreases in geriatric age.

## 5. Conclusions

In conclusion, our results support the possible use of deslorelin acetate as an adjuvant in the treatment of leishmaniasis in male dogs and, consequently, its possible use in the multimodal treatment of the disease.

However, further investigations are required to evaluate the efficacy of deslorelin acetate, comparing male unaltered dogs affected by CanL and females, to better understand the role of sex hormones in the development and outcome of the disease.

## Figures and Tables

**Table 1:** Clinical scores used to evaluate variables at day (D)0, D90 and D180 [6].

Clinical Signs	Scores			
	0	1	2	3
Appetite	Normal	Slight decrease	Moderate decrease	Anorexia
Mentation	Normal	Slight depression	Depression	Prostration
Exercise Intolerance	No	Slight	Moderate	Refusal to move
Weight Loss	No	Slight	Moderate	Severe
Polyuria	No	Slight	Moderate	Severe
Polydipsia	No	Slight	Moderate	Severe
Urine Protein/Urine Creatinine	No	<1	>1 < 2	>2
Localized Muscle Atrophy (Temporal Muscles)	No	Slight	Moderate	Severe
Generalized Muscle Atrophy	No	Slight	Moderate	Severe
Lymph Adenomegaly	No	1–2 nodes	>2 < 4 nodes	Generalized
Splenomegaly	No	-	Yes	-
Conjunctivitis and/or Blepharitis	No	Unilateral and slight	Bilateral or unilateral severe	Bilateral and severe
Uveitis and/or Keratitis	No	Unilateral and slight	Bilateral or unilateral severe	Bilateral and severe
Pale Mucous Membranes	No	Slight	Moderate	Severe
Epistaxis	Never presented	Sporadic	Frequent	Persistent
Mouth Ulcers or Nodules	No	1 or 2 small ulcers or nodules	>2 small ulcers or nodules	>1/4 of oral cavity covered by ulcers or nodules
Vomiting	No	Sporadic	Frequent	Frequent with blood
Diarrhoea	No	Sporadic	Frequent	Persistent
Lameness	No	Sporadic	Frequent	Constant
Erythema	No	<10% body surface or slight generalized erythema	10–25% body surface or moderate generalized erythema	>25% body surface
Dry Exfoliative Dermatitis	No	<10% body surface or slight generalized erythema	10–25% body surface or moderate generalized erythema	>25% body surface
Ulcerative Dermatitis	No	1–2 ulcers	3–5 ulcers	>5 ulcers
Nodular Dermatitis	No	1–2 nodules	3–5 nodules	>5 nodules
Sterile Pustular Dermatitis	No	1–2 pustules	3–5 pustules	>5 pustules
Alopecia	No	<10% body surface	10–25% body surface erythema	>25% body surface
Altered Pigmentation	No	Localized	Multifocal	Generalized
Hyperkeratosis Truffle and Pads	No	Slight	Moderate	Severe
Generalized Hyperkeratosis	No	Slight	Moderate	Severe

**Table 2:** Baseline characteristics of dogs included in the study expressed as mean ± standard deviation.

Variable	DES Group	CTR Group	*p*-Value
Age (Months)	58.7 ± 29.3	62.6 ± 18.1	0.94
Clinical Score (Points)	14.8 ± 5.8	11.2 ± 5.2	0.82
Body Temperature (°C)	39.4 ± 0.8	39.6 ±−0.81	0.46
Weight (kg)	18.6 ± 10.4	21 ± 8.4	0.79

**Table 3:** Mean ± standard deviation of clinical scores at D0, D90 and D180.

Time	Clinical Score	
Day	DES Group	CTR Group
D0	15.3 ± 6.3 ^ABC^	12 ± 4.9
D90	7.7 ± 5.9 ^ACa^	9.8 ± 4.3 ^Bb^
D180	2.1 ± 4.3 ^ABa^	5.2 ± 4.1 ^Ab^

Along the row, different lowercase letters (a and b) indicate significant differences in relation to the time; along the column, different capital letters (A, B and C) indicate significant differences in relation to the treatment. In the DES group at D90 and D180 was a significant difference of CS vs D0 (A > C, A > B and B > C). The CTR group showed a significant higher CS than DES group at D90 and D180 (a > b).

## Data Availability

Data that support the findings of this study are available on request from the corresponding author.
